# Engineering of inhalable nano-in-microparticles for co-delivery of small molecules and miRNAs

**DOI:** 10.1186/s11671-023-03781-0

**Published:** 2023-03-10

**Authors:** Marjan Motiei, Ondrej Mišík, Thanh Huong Truong, Frantisek Lizal, Petr Humpolíček, Vladimír Sedlařík, Petr Sáha

**Affiliations:** 1 Centre of Polymer Systems, University Institute, TBU, Tr. Tomase Bati, 5678 Zlin, Czech Republic; 2 Faculty of Mechanical Engineering, Brno University of Technology, Technicka 2896/2, 61669 Brno, Czech Republic

**Keywords:** Nano-in-microparticles, Pulmonary delivery, Chitosan, Small molecules, miRNAs

## Abstract

In this study, novel Trojan particles were engineered for direct delivery of doxorubicin (DOX) and miR-34a as model drugs to the lungs to raise local drug concentration, decrease pulmonary clearance, increase lung drug deposition, reduce systemic side effects, and overcome multi-drug resistance. For this purpose, targeted polyelectrolyte nanoparticles (tPENs) developed with layer-by-layer polymers (i.e., chitosan, dextran sulfate, and mannose-g-polyethyleneimine) were spray dried into a multiple-excipient (i.e., chitosan, leucine, and mannitol). The resulting nanoparticles were first characterized in terms of size, morphology, in vitro DOX release, cellular internalization, and in vitro cytotoxicity. tPENs showed comparable cellular uptake levels to PENs in A549 cells and no significant cytotoxicity on their metabolic activity. Co-loaded DOX/miR-34a showed a greater cytotoxicity effect than DOX-loaded tPENs and free drugs, which was confirmed by Actin staining. Thereafter, nano-in-microparticles were studied through size, morphology, aerosolization efficiency, residual moisture content, and in vitro DOX release. It was demonstrated that tPENs were successfully incorporated into microspheres with adequate emitted dose and fine particle fraction but low mass median aerodynamic diameter for deposition into the deep lung. The dry powder formulations also demonstrated a sustained DOX release at both pH values of 6.8 and 7.4.

## Introduction

The second most common carcinoma, lung cancer, is often treated with chemotherapy. However, effective cancer chemotherapy requires overcoming a number of drawbacks, including poor aqueous solubility, non-targeting ability, non-specific distribution, systemic side effects, a limited therapeutic index, and multi-drug resistance (MDR) [1, 2]. A practical approach to address these issues is simultaneous delivery of chemotherapeutics and non-chemotherapeutics, which possesses additional benefits like lowering drug dosages and achieving synergistic therapeutic efficacy [3, 4]. However, few studies utilized nanosystems for co-delivery of small molecules [5–7] and small molecules/tumor suppressor genes [8, 9]; herein, for the first time, co-delivery of model drugs such as doxorubicin (DOX) and miR-34a has been performed through targeted polyelectrolyte nanoparticles (tPENs) embedded in microparticles to combine the main beneficial characteristics of co-delivery of drugs, cell targeting ability, and low cytotoxicity.

Doxorubicin (DOX), as a leading anticancer drug, interacts with DNA and causes cancer cell apoptosis. This amphiphilic drug is effective against several cancer types, such as lung cancer [10]. miRNAs are a class of highly conserved single-stranded non-coding RNAs with fast biodegradation and a short half-life rate. They can inhibit the post-transcriptional gene expression by binding to 3′ untranslated regions of mRNAs [11]. miR-34a has become one of the most effective miRNAs as tumor suppressors, and its expression is downregulated in some human cancers, like lung cancer [12, 13]. However, such a strategy needs an efficient intracellular delivery system due to the fast biodegradation and short half-life of miRNAs [14, 15]. Therefore, the design of inhaled carriers with deep lung deposition and efficient intracellular co-delivery of DOX and miR-34a is the major challenge that needs to be overcome.

Pulmonary delivery is a promising method for lung cancer therapy because of the large alveolar surface area, thin epithelial barrier, extensive vascularization, absence of hepatic first-pass effect, and low enzymatic activity of the lungs [10]. The respirable dry formulations should have aerodynamic diameters from 1 to 5 µm to efficiently reach the deep lungs, swell upon deposition in the moist lung, and offer a sustained release through the matrix [16]. Particles larger than 5 µm would deposit in the upper airways primarily due to the inertial impaction [17]. Particles between 0.1 and 1 µm would be exhaled quickly, and particles smaller than 100 nm would be highly deposited in all airways, mainly in upper areas by the diffusional mechanism [18].

Strategically, nano-in-microparticles (NIMs) can improve the therapeutic effects by releasing nanoparticles (NPs) into the deep lungs and avoiding macrophage uptake. NPs can also anticipate as valuable platforms for targeted controlled release and degradation resistance of drugs [19]. Due to high biodegradability and biocompatibility [20], versatility in encapsulating hydrophilic and hydrophobic drugs, and capacity to cross biological barriers [21], polymeric NPs have attracted the most attention among the various types of NPs in inhaled therapeutic systems. Herein, a new class of therapeutic NPs was synthesized as tPENs composed of chitosan (CS) core and Mannose (Mans)-g-Polyethyleneimine (PEI) shell cross-linked electrostatically through negatively charged dextran sulfate (DS).

CS, a biocompatible, biodegradable, and antibacterial biopolymer, and PEI form stable nanocomplexes with polyanions such as tripolyphosphate (TTP) and DS through the protonation of amino groups below their pKa [22]. Due to the high expression of Mans receptors in human lung adenocarcinoma [23], and targeting both non-small cell lung cancer (NSCLC) cells and M2-like tumor-associated macrophages (M2-TAMs), PEI was functionalized with Mans [24]. Polyethyleneimine (PEI) also induces endosomal rupture by its “proton sponge effect” and facilitates the polyplexes’ release into cytosols [25]. After that, tPENs were micronized into a mucoadhesive excipient with low toxicity and readily degradable properties to increase the aerosolization efficiency and NPs’ uptake through the mucus layer [21]. CS, mannitol (Mant., a non-hygroscopic polymorphic acyclic sugar alcohol) [26], and leucine (Leu., hydrophobic amino acid) were the promising components of the excipient.

Moreover, this study has focused on the engineering and the feasibility of innovative biocompatible NIMs based on CS for the synergetic delivery of DOX and miR-34a in lung cancer therapy using a spray-drying technology. The innovative NIMs were developed to combine the benefits of smart multifunctional NPs and the respirable microspheres. The resulting micronized powders were fully characterized in terms of their morphology, production yield, flowability, aqueous reconstitution, and aerosolization efficiency, which confer a controlled and sustained release of cargos. Particle uptake into non-small-cell lung cancer was studied in A549 cells and compared to NIH-3T3 cell line at 2 h. Finally, the effect of DOX- and miR-34a-loaded tPENs was also evaluated on cytotoxicity.

## Result and discussion

### FT-IR analysis

**Fig. 1:**
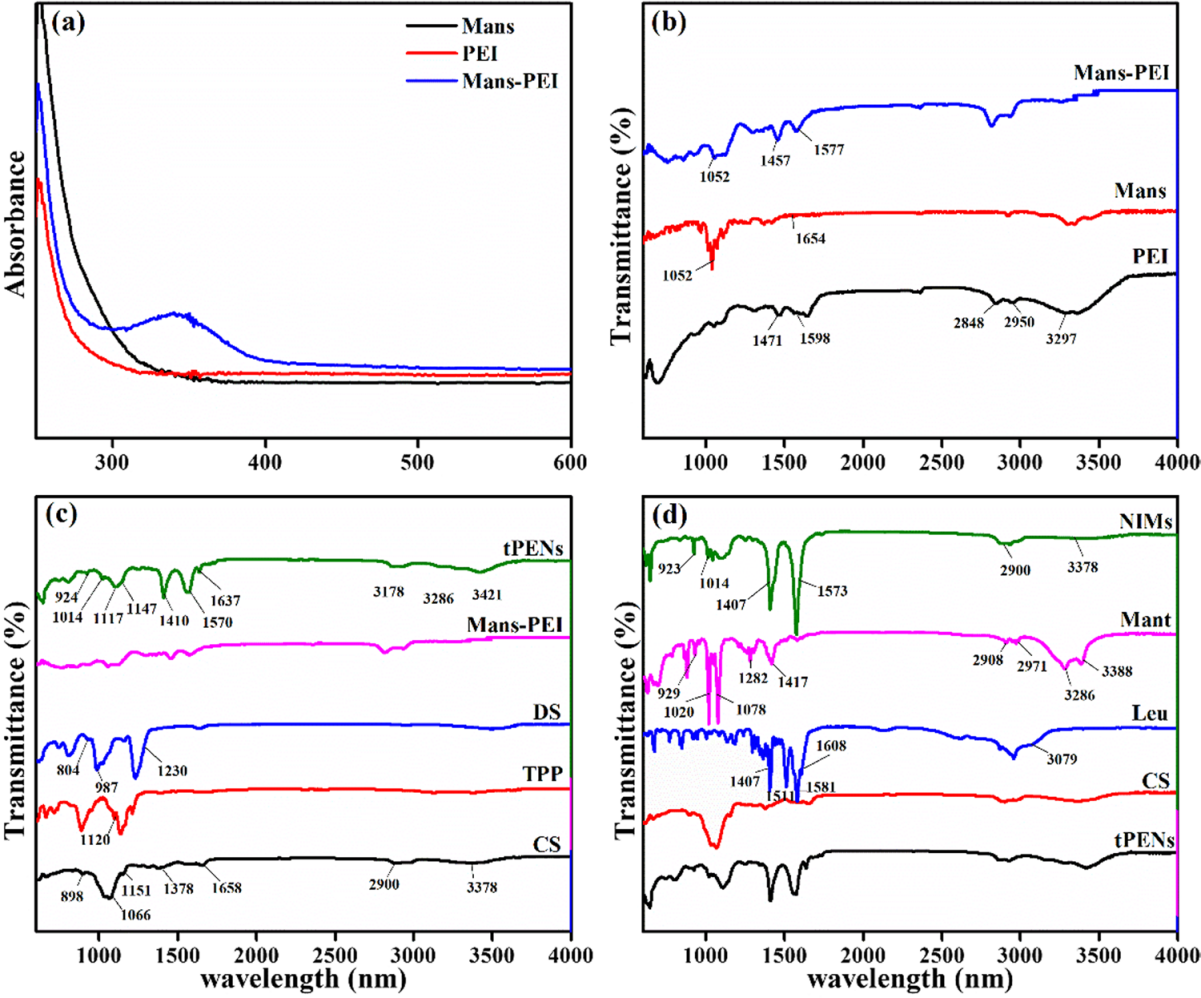
UV–Vis absorption spectra of PEI, Mans, and Mans-PEI (**a**), FT-IR spectra of PEI, Mans, and Mans-PEI (**b**) tPENs composed of CS, TPP, DS and Mans-PEI (**c**), and NIMs containing tPENs, CS, Leu, and Mant

A simple nucleophilic addition reaction between the primary amine of PEI and the aldehyde group of Mans was used to create Mans-PEI, and two distinct methods confirmed this. As shown in Fig. 1a, UV–Vis absorption spectra exhibited the modification of PEI with Mans by the presence of a new absorption peak at 340 nm as opposed to PEI and Mans [27]. To further explore the linkage formation between PEI and Mans, FT-IR was utilized. As shown in Fig. 1b, the characteristic absorptions of PEI at 2950, 2848, and 1471 cm^−1^ corresponded to the stretching and bending vibration of CH_2_ bonds, and the absorption peaks at 3297 and 1598 cm^−1^ belonged to the N–H bond [28]. Compared to the spectrum of PEI and Mans, the C=O stretching of Mans at 1654 cm^−1^ disappeared, and two strong absorption bands at 1457 and 1577 cm^−1^ were observed in the Mans-PEI spectrum assigned to the ring CC, and C-N stretches, respectively. In addition, the stretching vibration of C-O is located at 1052 cm^−1^, which reveals the presence of C–OH.

Figure 1c shows the FT-IR spectra of CS, TPP, DS, Mans-PEI, and tPENs. Two peaks in the FT-IR spectrum of CS powder were assigned at 898 and 1151 cm^−1^, which correspond to the structure of saccharides. The other peaks were assigned at 1066 cm^−1^ for the > CO-CH_3_ stretching vibration, 1378 cm^−1^ for the CH_3_ symmetrical deformation mode, 1658 cm^−1^ for the amide, 2900 cm^−1^ for the C-H stretching vibration, and 3378 cm^−1^ for the N–H symmetric stretching vibration. The bands at around 804 cm^−1^ obtained from the asymmetric S–O-S vibration and the asymmetric and symmetric SOO^−^ stretching vibrations at 1230 cm^−1^ and 987 cm^−1^ confirmed the presence of the sulfate group in the DS spectrum [29]. The tPENs’ spectra analysis revealed peaks at 1117 cm^−1^ and 1147 cm^−1^, which were associated with P=O in TPP [22] and the production of sulfones [30], respectively. Additionally, tPENs displayed sharp bands with high intensities at 924 cm^−1^, 1014 cm^−1^ (sulfo-group), 1410 cm^−1^ (symmetric stretching of COO −), 1570 cm^−1^ (Amide II band), 1637 cm^−1^ (C=O (amide I band)), 3286 cm^−1^ and 3178 cm^−1^ (O–H (H-bonded)), and 3421 cm^−1^ (primary amines). Therefore, it can be supposed that the polyplex has been achieved successfully.

In accordance with Fig. 1d, NH_3_^+^ vibrations are related to the spectra of Leu at 3079, 1608, and 1511 cm^−1^, and the consecutive bands at 1581 and 1407 cm^−1^ are attributed to the bending vibrations of the carboxyl group [31, 32]. The FT-IR spectrum of Mant shows strong stretching vibration peaks of OH (3286 and 3388), CO (1020 and 1078), and CH (2908 and 2971). Additionally, at 1417 and 1282 cm^−1^, the OH in-plane bending and CH bending vibrations can be seen [33]. When comparing the NIMs spectra to that of the other bulks, it can be demonstrated that several bands at 3079, 1608, and 1511 cm^−1^ have vanished, while peaks at 1407 and 1573 cm^−1^ associated with carboxyl vibrations have intensified, and peaks at 923, 1014, 2900, and 3378 cm^−1^ have appeared. Therefore, it can be supposed that CS, Leu, and Mant are preponderantly adsorbed into the NIMs.

### tPENs characterization

**Fig. 2:**
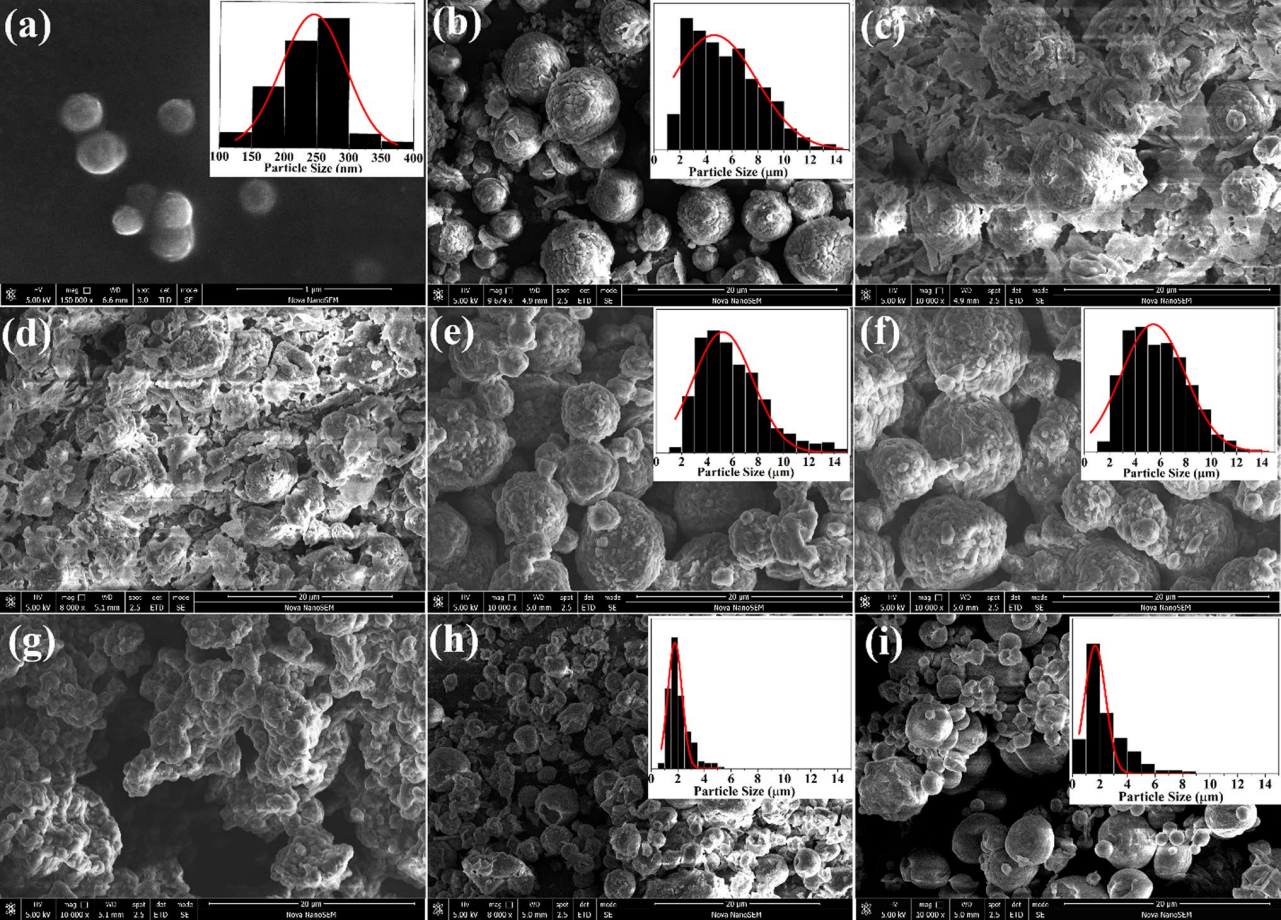
Size distributions and SEM images of tPENs (**a**) and different formulations of NIMs including F1 (**b**), F2 (**c**), F3 (**d**), F4 (**e**), F5 (**f**), F6 (**g**), F7 (**h**), and F8 (**i**)

SEM and DLS were used to describe the morphology, size, size distribution, and surface charge of the tPENs. Figure 2a indicates a SEM micrograph of uniform dispersed spherical tPENs with the size of 222.23 ± 54.88 nm. DLS analysis demonstrated that the hydrodynamic size of the tPENs was around 241.99 ± 0.51 nm with a relatively narrow distribution of 0.29 ± 0.01. The compact structure of the tPENs is attributed to pH, because the ionizable groups have a significant impact on the swelling behavior, network structure, and permeability of tPENs in response to pH fluctuation [34]. The presence of a sufficient positive charge on the tPENs’ surface (31.53 ± 4.21) also supports the colloidal stability of the nanostructures [35].

### NIMs characterization

Even though NPs have been suggested for effective drug delivery to the lung epithelium, the small size severely limits their application in pulmonary delivery because of high diffusional deposition (particle size ˂ 100 nm), exhalation (100 nm˂ particle size˂ 1 µm), or lung phagocytic clearance [36, 37]. Herein, different formulations of NIMs were synthesized in various ratios of excipients (i.e., CS, Leu, and Mant) to efficiently deliver therapeutic agents to the respiratory system and address these issues. F1, F2, and F3 were first synthesized in tPENs/CS ratios of 1/1, 1/1.5, and 1/3, respectively. According to Fig. 2b–d, the SEM micrographs showed that spherical particles with rough surfaces tend to be aggregated with increasing CS concentration. These deformations are probably caused by the spray-drying process and high repulsive forces among highly positive charged tPENs and CS. Therefore, F1 with a lower CS concentration and *d*_G_ of 5.90 ± 0.57 µm was selected for further experiments. After that, Leu was added to the excipient (i.e., F4, F5, and F6) to lessen NIM aggregation, delay the disintegration of the “interstitial bridges,” and decrease particle wetting [38, 39]. As shown in Fig. 2e–g, significant interparticle coalescences were demonstrated at higher Leu concentrations, and the physical properties of F6 were difficult to describe (not reported in Table 2). Some other studies also confirmed that crumpled particles are typical morphology of NIMs containing Leu [38]. Table 2 also demonstrates that the presence of Leu led to a significantly higher amount of Carr’s index and a lower amount of yield. Among the formulations F4-F6, F4 by Carr’s index of 21.25 ± 5.30 and yield of 40.13 ± 1.91 was selected for the subsequent development step.

In the next step, Mant was added to the F4 formulation to improve the viscoelasticity, liquid content, and aqueous re-dispersibility [40]. New formulations (F7 and F8) demonstrated that Mant significantly affected *d*_G_, *d*_A,theory_, and yield, which agrees with Kho & Hadinoto, who confirmed that Mant was the most promising candidate of NIMs morphology but not the aqueous re-dispersibility [37]. According to Fig. 2h, i, lower Mant concentration led to the formation of spherical particles with smaller size distribution. Finally, F7 was selected as an optimized formulation for further experiments due to significantly higher yields, re-dispersibility, and smaller *d*_G_ and *d*_A,theory_ (Table 1).

**Table 1:** Physical and aerosolization characteristics of inhaled dry powder

F	*ρ* eff (g/mL)	Carr’s index	Sf/Si	*d*_G_ (µm)	*d*_A_, _theory_	Yield (%)
F1	0.30 ± 0.02	15.22 ± 6.25^a^	1.63 ± 0.24	5.90 ± 0.57^a^	2.87 ± 0.68^a^	48.46 ± 8.54^a^
F4	0.32 ± 0.10	21.25 ± 5.30^b^	1.49 ± 0.06	5.26 ± 0.73^a^	2.67 ± 0.79^a^	40.13 ± 1.91^b^
F5	0.33 ± 0.05	20.68 ± 12.00^b^	1.54 ± 0.11	5.12 ± 1.22^a^	2.64 ± 0.84^a^	40.11 ± 11.04^b^
F7	0.33 ± 0.01	21.74 ± 7.59^b^	1.46 ± 0.09	2.09 ± 0.58^b^	1.07 ± 0.21^b^	49.65 ± 4.58^a^
F8	0.33 ± 0.03	23.08 ± 14.58^b^	1.97 ± 0.68	2.40 ± 0.98^b^	1.22 ± 0.34^b^	25.20 ± 4.25^c^

n = 3, Mean ± Standard Deviation. The different letters in the same column indicate significant differences between the means (*p* value < 0.05), and the values marked with the same letters are not statistically different

Due to the critical impact of particles’ aerodynamic behavior on lung deposition, the aerodynamic behavior of the two formulations (F4 and F7) was analyzed using APS and ACI. The average MMAD of F7, the breathable fraction of an aerosol, was in the range of 5.88 ± 0.57 and 6.42 ± 0.35 µm, and F4 was 5.87 ± 1.03 and 6.54 ± 0.05 µm by APS and ACI, respectively (Table 2). Most scientists strongly believe that particles larger than 5 µm should not be administered to the lungs [41]. However, in vivo measurements by Usmani et al. revealed that about 20% of 6 µm particles inhaled at a higher flow rate of 60 LPM were deposited in central and intermediate airways [42]. This is also in good agreement with one-dimensional computational models of dry powder lung delivery with idealized geometry replicas created by Finlay [43] and Soong et al. [44] at the inspiration flow rate of 70 LPM. These models predict that around 15% of 10 µm particles are deposited in tracheobronchial (TB), and the optimal TB drug delivery is attained by particles with MMAD of 2 to 5 µm when 25% to 55% of such particles are deposited in the TB area. Moreover, the powder formulations prepared in this work can be a suitable drug carrier for treating the TB region, especially in the first generations of branching.

**Table 2:** Aerodynamic behavior evaluated by APS and ACI

F		MMAD (µm)	GSD (µm)	FPF_<5 µm_ of APSD (%)	FPF_<5 µm_ of ED (%)	ED (%)
F4	APS	5.87 ± 1.03	1.74 ± 0.06	38.79 ± 13.98^a^	-	-
	ACI	6.54 ± 0.05	2.16 ± 0.04	30.30 ± 0.59	0.26 ± 0.00	90.50 ± 3.54
F7	APS	5.88 ± 0.57	1.58 ± 0.02	37.15 ± 8.53^b^	-	-
	ACI	6.42 ± 0.35	1.77 ± 0.15	33.11 ± 4.46	0,71 ± 0.00	96.17 ± 4.68

n = 3, Mean ± Standard Deviation. The different letters in the same column indicate significant differences between the means (*p* value < 0.05), and the values marked with the same letters are not statistically different

According to Table 2, the GSD of F7 and F4 is narrow, and 90.50 ± 3.54% of the applied dose from F4 and 96.17 ± 4.68% of F7 were dispersed into the impactor. FPFAPS and FPFACI also confirmed that more than 30% of the measured APSD were smaller than 5 µm. The actual deagglomeration causes the difference in FPFAPS and FPFACI within the Breezhaler device. The non-ideal deagglomeration shifts APSD to larger particles and significantly reduces the FPF. Therefore, these results confirmed that the final efficacy depends on the specific inhaler device and its deagglomeration ability and formulation properties. In the ACI measurements, the Breezhaler (Novartis) was used for aerosol dispersion, a device with quite a simple deagglomeration technology, low resistance, and ease to use [45].

**Fig. 3:**
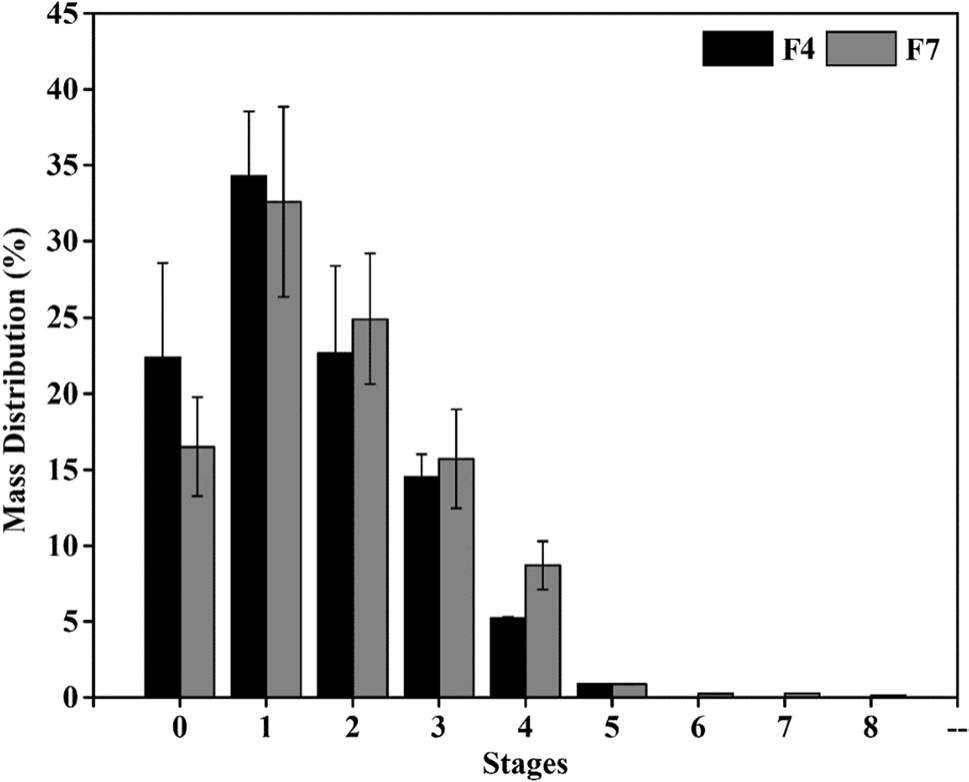
ACI deposition patterns of two spray-dried formulations (i.e., F4 and F7). Eight stages were analyzed fitting the particles cutoff diameter: stage 0 (˃8.6 µm), stage 1 (6.5–8.6 µm), stage 2 (4.4–6.5 µm), stage 3 (3.2–4.4 µm), stage 4 (1.9–3.2 µm), stage 5 (1.2–1.9 µm), stage 6 (0.55–1.2 µm), stage 7 (0.26–0.55 µm), and stage 8 (˂0.26)

Figure 3 displays the deposition patterns of the NIMs on an eight-stage ACI. A significant fraction of the aerosolized particles was collected in the mouthpiece adapter, the induction port, and the pre-separator, which signified the low FPF. However, the fraction of the aerosolized particles that ended up in stages 2, 3, and 4 were increased in F7 after adding Mant, confirming more effective agglomerate dispersion with slightly higher FPF than F4.

### Residual moisture content

To verify the effectiveness of the dry powder, TGA assessed the residual moisture content of the particles and excipients [46]. Physically and chemically preadsorbed CO_2_, moisture, and other gases are the main causes of the frequent weight loss below 100 °C. Therefore, thermogravimetric analysis of the samples was performed at 25–150 °C. At temperatures between 25 and 150 °C, Leu and Mant lost approximately 0.21% and 0.97% of their respective masses, and the moisture loss of tPENs was around 28.9% at a temperature of 61.66 °C. Nonetheless, the major moisture loss of NIMs (2.61%) occurred at 134.76 °C, which can be attributed to the hydrophobic Leu [47]. The chitosan’s hygroscopicity nature may lead to this high residual moisture [48], which results in additional size increases, lowering flowability, and reducing the long-term stability of DPI formulations [46]. It also affects the disposition of the drug particles at the point of deposition [49], and the ability to overcome the barriers related to lung geometry and the physiological conditions in the lungs [46].

### miR-34a and DOX loading efficiency and DOX in vitro release study

**Fig. 4:**
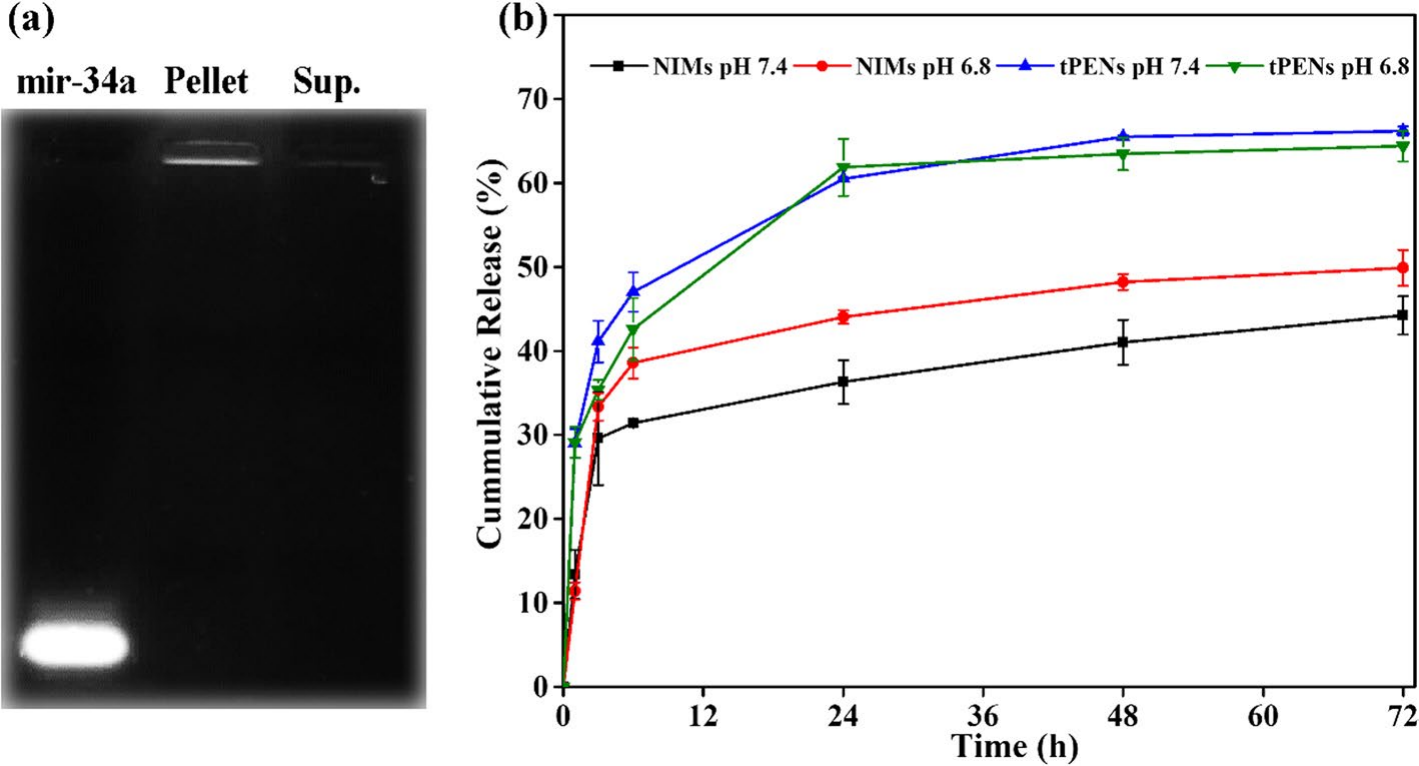
Gel retardation assay of mir-34a in tPENs at N/P ratio of 150 (**a**), DOX release profiles from tPENs and NIMs at 37 °C and two different pH values of 7.4 and 6.8 for 72 h. Each bar represents the average of three experiments, and error bars refer to standard deviation (**b**)

As demonstrated in Fig. 4a, in contrast to the other bands, which display a bright well with no trailing band for the pellets and no bright band for the supernatant, naked mir-34a, the negative control, exhibits a sharp band. Therefore, PENs interacted successfully with mir-34a and inhibit migration of mir-34a into the gel, in agreement with data already reported [50]. In quantification of DOX loading efficiency, tPENs demonstrated EE of 64.38 ± 0.61% and a lowering amount of LC (6.9 ± 0.24%), which can be explained by the strong dependency of LC on the weight ratio of tPENs in accordance with Eq. (6).

The cumulative release profile of both nano- and microparticles at two different pHs, physiological pH (7.4) and a more acidic pH (6.8) to mimic the lung-lining fluid of a lung cancer situation, is depicted in Fig. 4b. The results showed a similar release profile for the tPENs at pH 6.8 (64.38 ± 1.80%) and pH 7.4 (66.2 ± 0.54%) after 72 h, which can be discussed by high colloidal stability of tPENs [28]. For microformulation of F7, the DOX tends to release more sustainably than tPENs at both pHs, which is more pronounced at pH 7.4 (44.23 ± 2.27) than pH 6.8 (49.89 ± 2.13). It agrees with Lebhardt et al., who declared that larger particles demonstrate more sustained release in contrast to smaller ones because drug release relies on diffusion distance [40]. The higher drug release of NIMs at pH 6.8 can be explained by the pKa of CS (6.5) and its higher dissolution at pH 6.8 than 7.4. The prolonged-release profile, for at least 72 h, points out that the carriers improve the likelihood that the drug encounters the target for a longer time and releases it into the desired place.

### In vitro cytotoxicity of tPENs

**Fig. 5:**
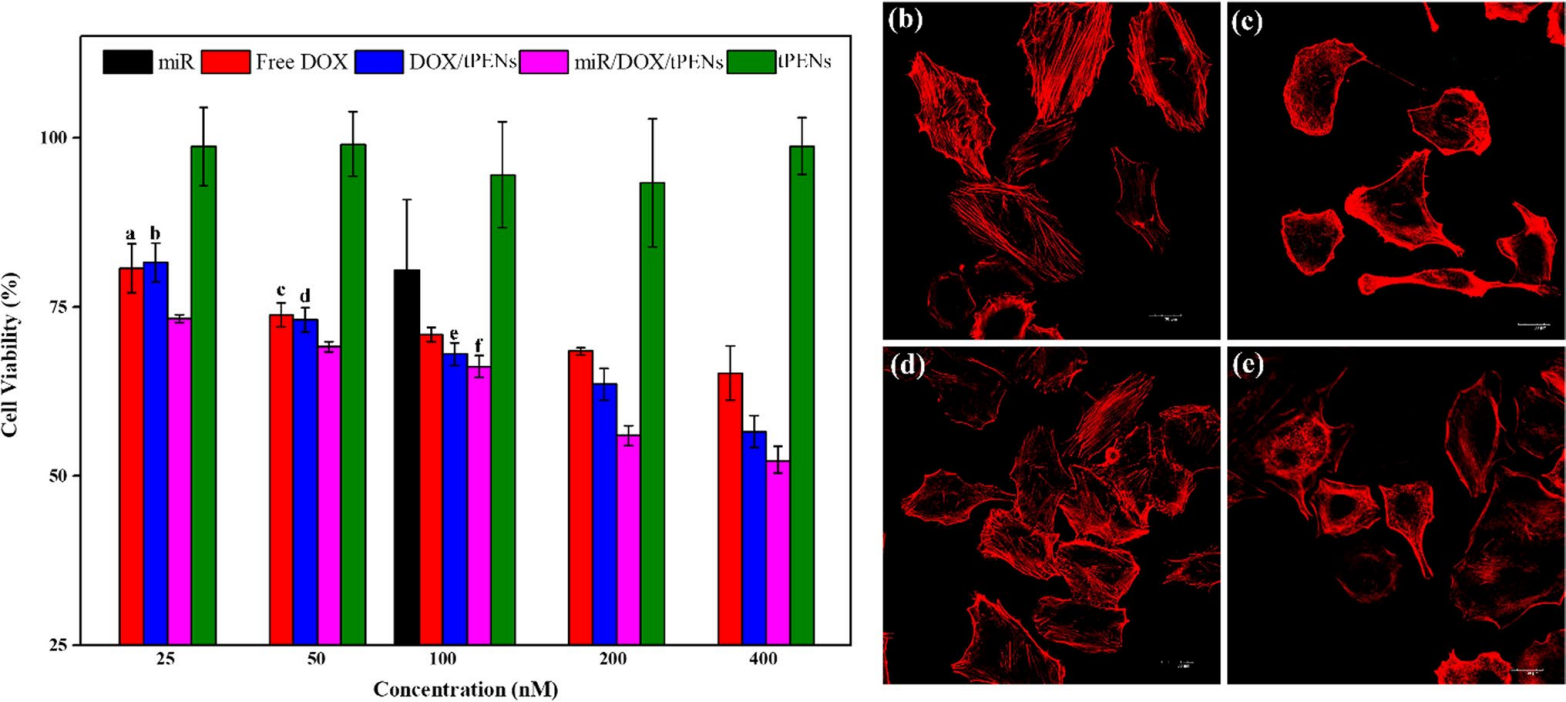
Cytotoxicity of tPENs, free drugs (i.e., DOX and miR-34a), and co-loaded tPENs against A549 cell line. The letters show *p* value greater than 0.05 in every concentration (**a**). Effects of tPENs (**b**), DOX (**c**), miR-34a (**d**), and co-loaded tPENs (**e**) on actin cytoskeleton of A549 cells. Images were acquired with × 60 objective, at identical exposure times, and scaled identically. Scale bars are 30 μm

Firstly, cytotoxicity studies were performed using tPENs, free drugs (i.e., DOX and miR-34a), and co-drugs-loaded tPENs against the A549 cell line by MTT assay. Human non-small cell lung cancer (NSCLC) cell line, A549, is a leading cause of cancer-related deaths worldwide and is typically an incurable disease [51]. According to Fig. 5a, there is no apparent toxicity by any concentration of tPENs, which makes them potentially safe and effective NPs. The MTT assay also revealed that free DOX triggered a significant cytotoxicity effect (65.28 ± 3.98) on the A549 cell line at the highest concentration (400 nM), which was significantly increased after loading in tPENs (56.60 ± 2.36, *p* value of 0.009). As depicted in Fig. 5a, 100 nM free miR-34a could reduce the viability of the A549 cell line by 20% over 24 h (80.39 ± 10.49). However, after co-treatment, a significant impact on the cell viability (52.29 ± 3.26) was observed at concentrations of 400 nM DOX and 100 nM miR-34a after 24 h. These results suggest that the cytotoxicity of DOX/miR-34a-loaded tPENs was more significant than DOX-loaded tPENs and also the free drugs.

After that, the effects of tPENs, DOX, miR-34a, and co-loaded tPENs on the actin cytoskeleton of A549 cells were assessed (Fig. 5). Therefore, the treated cells were stained by Alexa fluor 532-labeled phalloidin to show filamentous actin. Actin appeared more equally throughout the cell in tPENs-treated cells (Fig. 5b). As expected, DOX induced actin cytoskeleton remodeling with the formation of a cortical contractile ring at the cell periphery and the disruption of central stress fibers (Fig. 5c) inconsistent with Wei et al. [52]. In miR-34a treatment, the distribution of the actin cytoskeleton was partially unorganized and tended to form a cortical contractile ring at the cell periphery (Fig. 5d). Co-treatment with DOX and miR-34a for 24 h also induces cortical actin formation and reduction of stress fiber formation (Fig. 5e). Moreover, co-treatment with DOX and miR-34a simultaneously induces the disruption of central stress fibers and increases cortical actin formation.

### Cellular internalization of tPENs and PENs

**Fig. 6:**
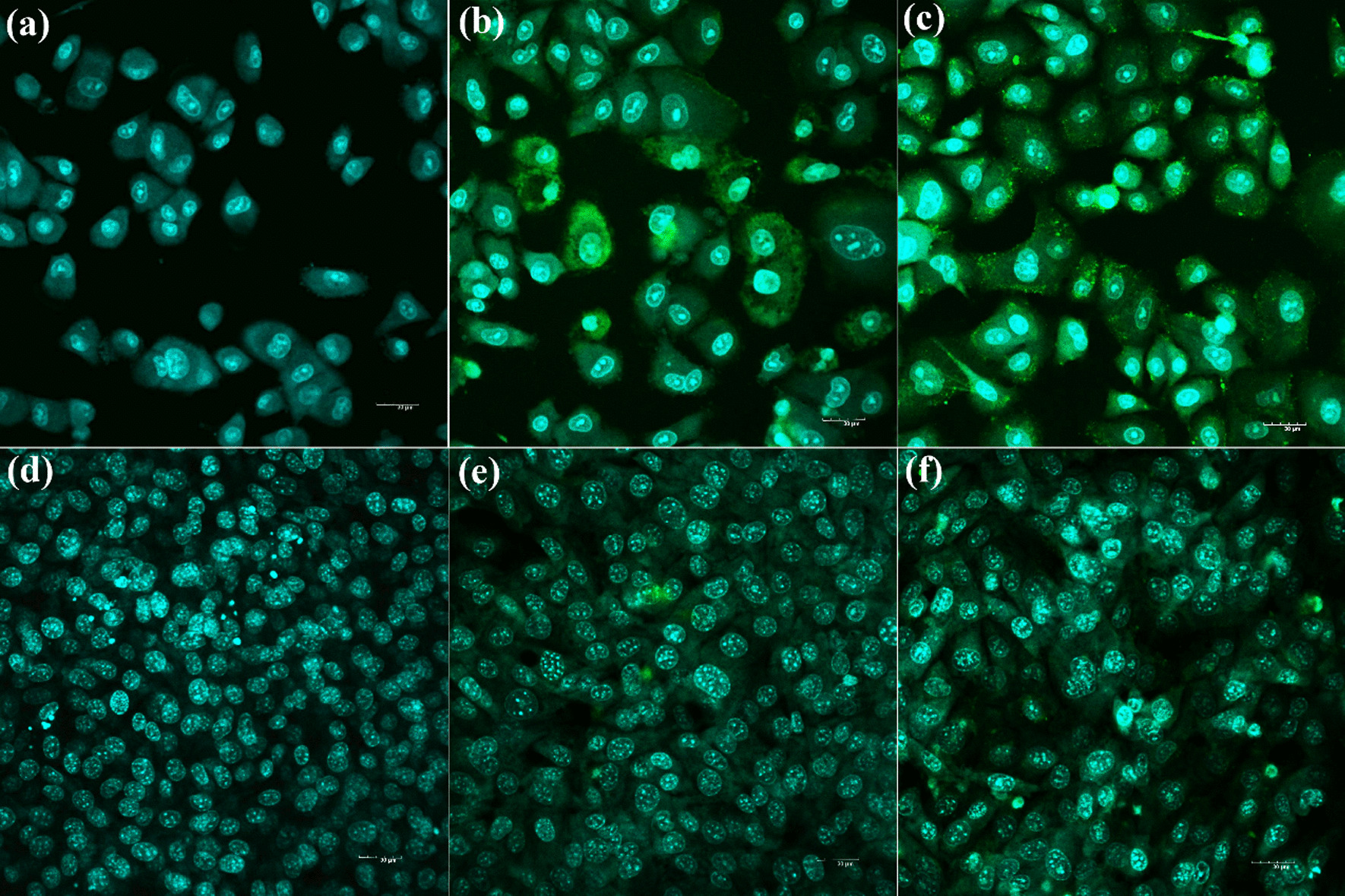
Confocal microscopy images of cellular internalization in untreated (**a**) and treated A549 cells with FITC-labeled PENs (**b**) and tPENs (**c**) in comparison with untreated (**d**) and treated NIH-3T3 cells by FITC-labeled PENs (**e**) and tPENs (**f**) after 2 h incubation at 37.0 °C

Targeting, passive or active, significantly impacts the therapeutic effects of NPs [53]. Drug efficacy and safety profiles of NPs can be enhanced by surface modifications in active targeting and physical features in passive targeting (i.e., size, functional groups, charge, and hydrophilicity of the surface) [53]. Herein, Mans has been utilized as a ligand with a high affinity to the Mans receptors as a prospective target for lung cancer therapy [23]. Therefore, the cellular uptake efficiency of labeled PENs and tPENs by FITC was assayed after 2 h incubation with A549, which expresses Mans receptors, and NIH-3T3 cell line as a negative control. In confocal microscopy images, the fluorescence of the labeled PENs was exhibited in the cytoplasm of the cells indicating the cellular internalization. According to Fig. 6b, c, tPENs showed comparable cell uptake levels to PENs in A549 cells; however, there was no discernible difference between tPENs and PENs in NIH-3T3 cells (Fig. 6e, f). In addition, A549 cells demonstrated significantly higher uptake levels than NIH-3T3 cells. Moreover, active targeting could increase cellular uptake by particular ligand-receptor interactions.

## Conclusion

The engineered formulation showed a significantly higher amount of Carr’s index and FPF, and a lower yield in the presence of leucine. Nonetheless, the inclusion of Mannitol was crucial to decrease *d*_G_ and *d*_A,theory_, and increasing yield. MMAD below 6 µm confirmed that the formulation could be a suitable drug carrier for treating the TB region, especially in the first generations of branching. NIMs tended to release DOX in a more sustained manner than tPENs at both pH values of 6.8 and 7.4. tPENs with high cellular uptake demonstrated no significant cytotoxicity on the metabolic activity of the A549 cell line, but co-loaded DOX/miR-34a onto tPENs showed greater cytotoxicity than DOX-loaded tPENs and free drugs using MTT assay and actin staining. On the whole, the results of this work suggest that the constructed nano-in-microparticles by spray-drying have a significant potential for the co-delivery of small molecules and miRNAs. Nonetheless, for developing this kind of therapy against cancer, in the subsequent study improving moisture content, average MMAD, and performing in vivo antitumor efficacy should be considered.

## Methods

### Materials

CS (MW of 50–190 kDa, DA: 15–25%), DS (MW of 7–20 kDa), PEI (MW of 1.3 kDa), polysorbate 80, Leu, Mant, Mans, doxorubicin hydrochloride, polydimethylsiloxane (PDMS), o-phthaldialdehyde (OPA), 2-mercaptoethanol, acetonitrile, sodium bicarbonate, dimethyl sulfoxide (DMSO), fluorescein isothiocyanate isomer I (FITC), cellulose dialysis tubing with cutoff 12 kD MWCO, and benzoylated dialysis tubing with cutoff 2000 NMWCO were purchased from Sigma Chemical Co. (St. Louis, MO, USA). miRIDIAN microRNA Mimics for precursor hsa-mir-34a-5p was purchased from PerkinElmer. Non-small human adenocarcinoma epithelial cell line (A549) and mouse embryonic fibroblast cell line NIH/3T3 (ATCC CRL-1658TM) were obtained from the European Collection of Authenticated Cell Cultures (ECACC). Roswell Park Memorial Institute (RPMI) 1640 Medium, Dulbecco’s Modified Eagle Medium (DMEM), Trypsin-EDTA, and fetal bovine serum (FBS) were purchased from Biosera (France). Antibiotic–antimycotic and MTT were prepared from Biowest (USA) and Duchefa (Biochemie, Netherlands), respectively. All other chemicals used in the study were of analytical grade.

### Synthesizing and characterization of tPENs

The Mans-PEI was first prepared according to a previously reported procedure [27, 54]. In brief, 0.04 g/mL Mans in PBS buffer (10 mM, pH 7.4) was added to 0.07 g/mL PEI in PBS. After heating the mixture at 90 ℃ for 1 h in a water bath, the solution was purified with a 1000 Da cutoff dialysis bag against dH_2_O for more than 24 h. The product inside the dialysis bag was collected for further analysis. For preparation of DOX loading tPENs, 84 µL DOX solution (1 mg/mL in DMSO/dH_2_O) was added to 3 mL CS solution (1 mg/mL in 1% acetic acid, 0.5% polysorbate 20, pH 5) under gentle stirring for 30 min. After that, 1 mL TPP (1 mg/mL, pH 8), 400 µL DS (1 mg/mL, pH 8), and then 300 µL Mans-PEI (10 mg/mL, pH 8) were added dropwise to complete tPENs synthesizing. The morphology of dried tPENs on aluminum foil was evaluated by SEM (Nova 450 NanoSEM, FEI, Brno, Czech Republic) operating at 5.00 kV accelerating voltage after sputter-coating by gold/palladium (SC7620 Mini Sputter Coater, Quorum Technologies,10 mA for 45 s). Particle size and zeta potential of tPENs were also measured by DLS (model 3600, Malvern Instruments Ltd., Worcestershire, UK).

### Synthesizing of NIMs by spray-drying

As shown in Table 3, NIMs were synthesized in tPENs/CS *w*/*w ratio* of 1/1 (F1), 1/1.5 (F2) and 1/3 (F3). *w*/*w ratio* of 1 was selected for the next formulations (i.e., F4, 5, 6, 7, 8) in different ratios of excipients containing CS, Leu, and Mant. The excipients were dissolved in acetic acid 1%, filtrated, and adjusted pH to 5. After that, the tPENs were added to the excipient solution under stirring for 15 min to be homogenized. The mixture was fed to a BUCHI B-290 mini spray dryer (BÜCHI, Switzerland) operated in an open-loop mode using compressed air as the drying gas. The spray-drying apparatus employed a two-fluid nozzle. The air spray flow and aspirator rate were kept constant at 250 l/h and 100%, respectively, with the inlet temperature of 90 ͦ C and outlet temperature of 35 ͦ C. The total solid concentration in the feed was around 5 mg/mL.

**Table 3:** Different formulations of NIMs

	Formulations	tPENs/excipient ratio	Excipient		
			CS	Leu	Mant
NIMs	F1	1/1	10	0	0
	F2	1/1.5	15	0	0
	F3	1/3	30	0	0
	F4	1	9	1	0
	F5	1	7	3	0
	F6	1	5	5	0
	F7	1	9	0.9	0.1
	F8	1	9	0.8	0.2

NIMs were synthesized in tPENs/excipient w/w ratio of 1, 1/1.5, and 1/3. w/w ratio of 1 was selected for the next formulations (F4,5,6,7,8) in different ratio of excipients

### Powder characterizations

The NIMs powders were stored at 4 ͦ C before characterizations in terms of morphology, geometric diameter (*d*_G_), aerodynamic diameter (*d*_A,theory_), effective density (*ρ*_eff_), yield, flowability, and reconstitution. NIMs morphology was characterized by SEM and *d*_G_ by the SEM images of 1000 particles using ImageJ software (NIH, USA). *ρ*_eff_ was determined by the tap density (*ρ*_tap_) measured by a tap density analyzer (Quantachrome Autotap) after 2000 taps corrected by a factor of 0.79^−1^ to consider the imperfect particle packing [37]. The *d*_A,theory_ was calculated from Eq. (1), where *ρ*_S_ is 1 g/cm^3^ [20].

1 $$d_{{\text{A}}} \;{\text{theory}} \left( {\upmu {\text{m}}} \right) = d_{{\text{G}}} \sqrt {\rho_{{{\text{eff}}}} /\rho_{{\text{s}}} }$$

The mass ratio of the collected powders to the total solids was used to compute the production yield. The powder flowability was characterized by Carr’s index calculated from Eq. (2), where *ρ*_tap and_ *ρ*_bulk_ are the particle densities before and after tapping. Carr’s index ≤ 25 shows free-flowing, and Carr’s index ≥ 40 illustrates poor-flowing.

2 $${\text{Carr's}}\;{\text{index}} \;\left( \% \right) = \left( {1 - \frac{{\rho_{{{\text{bulk}}}} }}{{\rho_{{{\text{tap}}}} }}} \right) \times 100$$

NIMs reconstitution in an aqueous medium is characterized by changes in the NPs’ size before and after reconstitution. Briefly, 5 mg of NIMs was added to 3 mL of dH_2_O (pH 6.8) under gentle stirring for 15 min. Then, centrifugation at 6000 rpm for 10 min was followed by collecting the supernatant to determine Sf using DLS. When the ratio of the averaged sizes of reconstituted tPENs to raw tPENs (Sf/Si) is around 1, it denotes complete reconstitution, whereas Sf/Si ratio > 1 denotes poor reconstitution.

### Powder dispersion and sizing by Aerodynamic Particle Sizer and Andersen Cascade Impactor

The cascade impactor and time-of-flight sizer are two techniques used to assess particle size distributions. Cascade impactors with an induction port are used in the compendial technique outlined in the United States Pharmacopeia/European Pharmacopeia (USP/Ph. Eur.), simulating the upper airways’ 90° bend [55]. This method explains the size distribution of the powder along with the unique characteristics of the inhaler. But if the properties of the formulation must be specified, a time-of-flight particle spectrometer is preferable in which deagglomerated particles are supplied by a dedicated powder disperser.

The aerodynamic particle size distribution (APSD) of selected formulations (F4, F7) was determined by Aerodynamic Particle Sizer 3321 (APS, TSI, Minnesota, USA). The aerosol was dispersed by Small-Scale Powder Disperser 3433 (SSPD, TSI) and delivered into APS via an electroconductive hose. The average size distribution of ten measurements was used to calculate the mass median aerodynamic diameter (MMAD) of each powder sample. In order to assess the impact of realistic dispersing circumstances on the chosen formulations, an eight-stage Andersen Cascade Impactor (ACI, Copley, UK) was used.

In ACI analysis, ten capsules no. 3 containing 20 mg of NIMs were inserted into a Breezhaler (Novartis), a single dosage inhaler, and connected to the ACI inlet. To mimic inhalation, the capsule was ruptured, and 4 L of air was pumped through it at a steady flow rate of 60 L/min for 4 s. Particles were separated and sorted according to their aerodynamic diameter in the eight impactor collection plates. A thin layer of PDMS was applied to the inner surfaces of the induction port and pre-separator to prevent aerosol particles from re-bouncing. The recovered powder on the cascade stages and the filter were dispersed in 20 ml of 1% acetic acid in the presence of an ultrasonic bath for 5 min. After that, the solutions were tested for the parameters including emitted dose (ED), fine particle fraction (FPF), MMAD, and geometric standard deviation (GSD) [1]. HPLC was used to estimate the Leu’s mass deposited on each stage, and then, the normalized cumulative APSD was computed. The mass proportion of the powders that successfully eject from the inhaler and are retrieved in the ACI is represented by ED. According to Eq. (3), M Full, M Empty, and M Powder represent the capsule weights before and after inhalation and the starting weight of powder in the capsule, respectively.

3 $${\text{ED }}\left( {\text{\%}} \right) = \left( {\frac{{{\text{M}}\;{\text{full}} - {\text{M}}\;{\text{empty}}}}{{{\text{M}}\;{\text{powder}}}}} \right) \times 100$$

FPF was calculated as a percentage of the total mass of APSD made up of particles smaller than 5 m. GSD was calculated using Eq. (4), where d16 were the diameters corresponding to 16% of the cumulative distribution, and MMAD was defined as the particle diameter corresponding to 50% of the cumulative distribution.

4 $${\text{GSD}} \left( \% \right) = \frac{{{\text{MMAD}}}}{{d_{16} }}$$

### Moisture content

A TA Instruments Q500 Thermogravimetric Analyzer was used to ascertain the residual moisture content in the spray-dried NIMs between 25 and 900 °C at a heating rate of 10 °C/min. Solid samples (5–10 mg) were loaded on open platinum TGA pan, and the moisture content (water loss) was analyzed using the Universal Analysis 2000 system between 25 to 150 °C [46].

### Quantification of miR-34a and DOX loading efficiency and DOX in vitro release study

The gel retardation assay at a N/P ratio of 150:1 was used to assess the miR-34a loading efficiency of the tPENs [50, 56]. Briefly, the vortexed miR-34a-loaded Mans-PEI was added dropwise to the core solution, vortexed for 6 s, and then incubated for 30 min at RT. tPENs solution was centrifuged at 13,000 rpm for 15 min, and the supernatant and pellet were both analyzed. 18 µL of polyplex solution was analyzed on 2% agarose gel containing Red Safe after the solutions were mixed with 2µL of 6 $\times$ loading buffer. Gel electrophoresis was performed using a Sub-Cell system (Bio-Rad Laboratories, CA) in 1 TAE buffer at 120 V for 15 min, and miRNA bands were detected using a GelDoc system (UVITEC, UK).

After that, loading efficiency of DOX-loaded tPENs was evaluated by dialysis technique and in the presence of PBS 10 mM, pH 7.4. Briefly, the dispersions were introduced into a 100 mL PBS (10 mM, pH 7.4) by a 12 MWCO dialysis tube, and then, the entire system was kept in an orbital incubator (Stuart SI500, UK) at 37 ± 0.5 °C, 40 rpm for 1 h. Encapsulation efficiency (EE) and loading capacity (LC) were calculated according to Eqs. (5) and (6), respectively [53], where total DOX was the amount of primary DOX added to tPENs solution, and free DOX was evaluated by HPLC.

5 $${\text{EE}}\;\left( {\text{\%}} \right) = \frac{{{\text{Total}}\;{\text{DOX}} - {\text{Free}}\;{\text{DOX}}}}{{{\text{Total}}\;{\text{DOX}}}} {\times} 100$$

6 $${\text{LC }}\;\left( {\text{\%}} \right) = \frac{{{\text{Total}}\;{\text{DOX}} - {\text{Free}}\;{\text{DOX}}}}{{{\text{Total}}\;{\text{Weight}}\;{\text{of}}\;{\text{Particles }}}} {\times} 100$$

Afterward, to evaluate the in vitro release rate of tPENs, the medium was changed with 50 mL PBS 10 mM (pH 7.4 and 6.8), and the entire system was maintained in an orbital incubator at 37 ± 0.5 °C, 40 rpm. For evaluation of DOX-loaded NIMs release, 100 mg of the NIMs was introduced into a 12 MWCO dialysis membrane, and then, the membrane was suspended in the buffer solution. At pre-determined time intervals, 2 mL of the medium was taken, and the same volume of the fresh medium was replaced in the system. HPLC was used to assess the quantity of DOX present in the medium.

### HPLC

HPLC analysis of Leu and DOX was carried out by a Thermo Scientific Dionex UltiMate 3000 (Germany) on an XSELECT CSH C18 reversed-phase column, 5 µm, 4.6 × 250 mm (Waters, USA) with a security guard (Phenomenex, USA). For Leu analysis, pre-column derivatization was performed by OPA solution. The derivatization reagent was made up of a variety of solutions, such as buffer solution (5% H_3_BO_3_ in water, pH 11 with KOH 47%), reducing solution (2.5% 2-mercaptoethanol in buffer solution), and OPA solution (2.5 mg OPA + 400 µl methanol + 200 µl reducing solution + 4.4 ml buffer solution) [57]. The filtered samples were placed in the autosampler at 7 °C. The volume of 10 µl was injected into the column originating from a drawn sample volume of 1 µl. A gradient of 100 mM acetate buffer (A; pH 5.8) and HPLC grade acetonitrile (ACN; B) at a flow rate of 0.4 ml/min made up the mobile phase. The fluorescently active OPA derivatives were detected at *ƛ*Ac = 330 nm and *ƛ*em = 440 nm, respectively, by an external calibration approach using the following calibration curve equation: *y* = 24163*c* + 614,75 (*R*^2^ = 0.9999; concentration from 0.1–10). y represents the peak area, and c the concentration.

In the HPLC analysis of DOX, a mixture of 20 mM KH_2_PO_4_ buffer and HPLC grade acetonitrile was utilized as the mobile phase (65:35, v/v) at a flow rate of 1.0 ml/min. The sampler was adjusted at 7 °C, the length of the isocratic run was 8 min, and the injection volume was 20 µl. As excitation and emission wavelengths, the eluted DOX was detected fluorescently at 480 nm and 550 nm. The external calibration method was utilized to quantify DOX using the following calibration curve equation: *y* = 4339.3*c* − 748.36 (*R*^2^ = 0.9793; concentration from 0.25 to 5 µg/ml). *y* represents the peak area, and c the concentration.

### In vitro cytotoxicity of particles

A549 cell line was seeded in RPMI1640 medium supplemented with 10% v/v FBS and antibiotic antimycotic solution (penicillin G, 100 U mL^−1^ and streptomycin G, 100 μg mL^−1^) in a humidified atmosphere (95% air and 5% CO_2_) at 37ͦC. To assess the cytotoxicity effect of tPENs, DOX, miR-34a, DOX-loaded tPENs, and miR-34a/DOX-loaded tPENs, the cells were subcultivated in Trypsin-EDTA solution 0.25% for 3 min, and then, the cells at the concentration of 1 × 10^5^ per mL were grown in a 96-well plate for 24 h. After the adhesion phase, the cells were treated with 100 μL of the medium containing different concentrations of free DOX (400, 200, 100, 50, and 25 nM), free miR-34a (100 nM), tPENs, and tPENs loading DOX and DOX/miR-34a. After 24 h incubation, a standard MTT assay was performed using 100 μL of RPMI1640 containing 10% MTT for 4 h and 80 μl of solubilization buffer (DMSO) for 15 min at 37 °C in a CO_2_ incubator. Luminometer Infinite M200 Pro NanoQuant (Tecan, Switzerland) at 570 nm and the reference wavelength of 690 nm was utilized for measuring the cell viability. The effect of tPENs, DOX-loaded, miR-34a-loaded, and DOX/miR-34a-loaded tPENs on the actin cytoskeleton of A549 cells was also evaluated. For this purpose, the cells were permeabilized with 0.5% triton for 5 min after being fixed with 4% paraformaldehyde for 15 min. Thereafter, f-actin was labeled by Alexa fluor-532-labeled phalloidin (30 min) and stored in the dark before imaging by confocal laser scanning microscopy (Olympus, Japan).

### Cellular internalization

The PENs were FITC-labeled to be visualized the intracellular localization. Briefly, PEI and Mans-PEI were dissolved in dH2O (10 mg/mL), adjusted pH to 11, and then, FITC was added under stirring overnight. Following dialysis, the labeled PEI and Mans-PEI were utilized as a shell in PENs and tPENs structure. After that, A549 and NIH-3T3 were grown in RPMI1640 and DMEM media, respectively, supplemented with 10% v/v FBS and antibiotic–antimycotic on 12 Well glass bottom plate at 37ͦC, under a 5% CO2 humidified atmosphere, for 24 h. After washing the cells with PBS, they were treated with a solution of medium containing labeled NPs (1 mg/mL). Two hours later, the cells were washed with PBS pH 7.4 to remove non-uptaken NPs. After staining the nucleus of the formaldehyde-fixed triton-permeabilized cells with Hoechst (30 min), they were evaluated using Leica SP8 confocal microscopy.

### Statistical analysis

The data were provided in triplicate and as mean ± standard deviation. IBM SPSS Statistics 26 and Microsoft Excel 2010 were utilized to conduct the statistical analysis. The mean values were compared using one- and two-way analyses of variance (ANOVA), and at a *p* value of 0.05, the differences were declared significant.

## Data Availability

All data generated or analyzed during this study are included in this published article.

## Abbreviations

*d*_A,theory_: Aerodynamic diameter
APSD: Aerodynamic particle size distribution
APS: Aerodynamic Particle Sizer
ACI: Andersen Cascade Impactor
CS: Chitosan
DS: Dextran sulfate
DMSO: Dimethyl sulfoxide
DOX: Doxorubicin
DMEM: Dulbecco’s Modified Eagle Medium
*ρ*_eff_: Effective density
ED: Emitted dose
EE: Encapsulation efficiency
ECACC: European Collection of Authenticated Cell Cultures
FBS: Fetal bovine serum
FPF: Fine particle fraction
FITC: Fluorescein isothiocyanate isomer I
*d*_G_: Geometric diameter
GSD: Geometric standard deviation
NSCLC: Human non-small cell lung cancer
Leu: Leucine
LC: Loading capacity
Mant: Mannitol
Mans: Mannose
MMAD: Mass median aerodynamic diameter
M2-TAMs: M2-like tumor-associated macrophages
NIH/3T3: Mouse embryonic fibroblast cell line
MDR: Multi-drug resistance
NPs: Nanoparticles
NSCLC: Non-small cell lung cancer
A549: Non-small human adenocarcinoma epithelial cell line
OPA: O-phthaldialdehyde
PDMS: Polydimethylsiloxane
PEI: Polyethyleneimine
RPMI: Roswell Park Memorial Institute
SSPD: Small-Scale Powder Disperser
*ρ*_tap_: Tap density
tPENs: Targeted polyelectrolyte nanoparticles
TB: Tracheobronchial
TPP: Tripolyphosphate
USP/Ph. Eur.: United States Pharmacopeia/European Pharmacopeia

## References

[1] Ding Y, Liu W, Yu W, Lu S, Liu M, Kaplan DL (2018). Three-dimensional tissue culture model of human breast cancer for the evaluation of multidrug resistance. J Tissue Eng Regen Med.

[2] Motiei M, Aboutalebi F, Forouzanfar M, Dormiani K, Nasr-Esfahani MH, Mirahmadi-Zare SZ. Smart co-delivery of miR-34a and cytotoxic peptides (LTX-315 and melittin) by chitosan based polyelectrolyte nanocarriers for specific cancer cell death induction. Mater Sci Eng C. 2021:112258. 10.1016/j.msec.2021.112258 34474818

[3] Gandham SK, Rao M, Shah A, Trivedi MS, Amiji MM (2022). Combination microRNA-based cellular reprogramming with paclitaxel enhances therapeutic efficacy in a relapsed and multidrug-resistant model of epithelial ovarian cancer. Mol Ther Oncolytics.

[4] Lo Y-L, Lin H-C, Tseng W-H (2022). Tumor pH-functionalized and charge-tunable nanoparticles for the nucleus/cytoplasm-directed delivery of oxaliplatin and miRNA in the treatment of head and neck cancer. Acta Biomater.

[5] Xiao J, Weng J, Wen F, Ye J. Red blood cell membrane-coated silica nanoparticles codelivering DOX and ICG for effective lung cancer therapy. ACS Omega 2020. 10.1021/acsomega.0c01541 PMC7774068 33403246

[6] Han W, Shi L, Ren L, Zhou L, Li T, Qiao Y (2018). A nanomedicine approach enables co-delivery of cyclosporin A and gefitinib to potentiate the therapeutic efficacy in drug-resistant lung cancer. Signal Transduct Target Ther.

[7] Shen Y, TanTai J (2020). Co-delivery anticancer drug nanoparticles for synergistic therapy against lung cancer cells. Drug Des Dev Ther.

[8] Liu J, He J, Zhang M, Xu G, Ni P (2018). A synergistic polyphosphoester-based co-delivery system of the anticancer drug doxorubicin and the tumor suppressor gene p53 for lung cancer therapy. J Mater Chem B.

[9] Taratula O, Kuzmov A, Shah M, Garbuzenko OB, Minko T (2013). Nanostructured lipid carriers as multifunctional nanomedicine platform for pulmonary co-delivery of anticancer drugs and siRNA. J Control Release.

[10] Xu C, Wang Y, Guo Z, Chen J, Lin L, Wu J (2019). Pulmonary delivery by exploiting doxorubicin and cisplatin co-loaded nanoparticles for metastatic lung cancer therapy. J Control Release.

[11] Zhang L, Liao Y, Tang L (2019). MicroRNA-34 family: a potential tumor suppressor and therapeutic candidate in cancer. J Exp Clin Cancer Res.

[12] Li J, Che L, Xu C, Lu D, Xu Y, Liu M, et al. XIST/miR-34a-5p/PDL1 axis regulated the development of lung cancer cells and the immune function of CD8+ T cells. J Recept Signal Transduct. 2022:1–10. 10.1080/10799893.2021.2019274 35067156

[13] Abtahi NA, Naghib SM, Ghalekohneh SJ, Mohammadpour Z, Nazari H, Mosavi SM (2022). Multifunctional stimuli-responsive niosomal nanoparticles for co-delivery and co-administration of gene and bioactive compound: In vitro and in vivo studies. Chem Eng J.

[14] Trivedi M, Singh A, Talekar M, Pawar G, Shah P, Amiji M (2017). MicroRNA-34a encapsulated in hyaluronic acid nanoparticles induces epigenetic changes with altered mitochondrial bioenergetics and apoptosis in non-small-cell lung cancer cells. Sci Rep.

[15] Sharma P, Dando I, Strippoli R, Kumar S, Somoza A, Cordani M, et al. Nanomaterials for autophagy-related miRNA-34a delivery in cancer treatment. Front Pharmacol. 2020;11. 10.3389/fphar.2020.01141 PMC7393066 32792960

[16] Silva AS, Sousa AM, Cabral RP, Silva MC, Costa C, Miguel SP (2017). Aerosolizable gold nano-in-micro dry powder formulations for theragnosis and lung delivery. Int J Pharm.

[17] Darquenne C (2020). Deposition mechanisms. J Aerosol Med Pulm Drug Deliv.

[18] Frederix EMA, Kuczaj AK, Nordlund M, Belka M, Lizal F, Jedelsky J (2018). Simulation of size-dependent aerosol deposition in a realistic model of the upper human airways. J Aerosol Sci.

[19] Restani RB, Silva AS, Pires RF, Cabral R, Correia IJ, Casimiro T (2016). Nano-in-micro POxylated polyurea dendrimers and chitosan dry powder formulations for pulmonary delivery. Part Part Syst Charact.

[20] Wang Y, Kho K, Cheow WS, Hadinoto K (2012). A comparison between spray drying and spray freeze drying for dry powder inhaler formulation of drug-loaded lipid–polymer hybrid nanoparticles. Int J Pharm.

[21] Ruge CA, Bohr A, Beck-Broichsitter M, Nicolas V, Tsapis N, Fattal E (2016). Disintegration of nano-embedded microparticles after deposition on mucus: a mechanistic study. Colloids Surf B.

[22] Motiei M, Kashanian S (2017). Novel amphiphilic chitosan nanocarriers for sustained oral delivery of hydrophobic drugs. Eur J Pharm Sci.

[23] Yin L, Chen Y, Zhang Z, Yin Q, Zheng N, Cheng J (2015). Biodegradable micelles capable of mannose-mediated targeted drug delivery to cancer cells. Macromol Rapid Commun.

[24] Jaynes JM, Sable R, Ronzetti M, Bautista W, Knotts Z, Abisoye-Ogunniyan A, et al. Mannose receptor (CD206) activation in tumor-associated macrophages enhances adaptive and innate antitumor immune responses. Sci Transl Med. 2020;12(530):eaax6337. 10.1126/scitranslmed.aax6337 PMC7832040 32051227

[25] Wang W, Li W, Wang J, Hu Q, Balk M, Bieback K, et al. Folate receptor mediated genetic modification of human mesenchymal stem cells via folic acid-polyethylenimine-grafted poly (N-3-hydroxypropyl) aspartamide. Clin Hemorheol Microcirc. 2017(Preprint):1–17. 10.3233/CH-179209 28869460

[26] De Pauw E, Vervaet C, Vanhoorne V (2022). Formation of delta-mannitol by co-spray drying: enhancing the tabletability of paracetamol/mannitol formulations. J Drug Deliv Sci Technol.

[27] Liu M, Li J, Li B (2018). Mannose-modificated polyethylenimine: a specific and effective antibacterial agent against Escherichia coli. Langmuir.

[28] Motiei M, Sedlařík V, Lucia LA, Fei H, Münster L (2020). Stabilization of chitosan-based polyelectrolyte nanoparticle cargo delivery biomaterials by a multiple ionic cross-linking strategy. Carbohydr Polym.

[29] Chavan C, Bala P, Pal K, Kale S (2017). Cross-linked chitosan-dextran sulphate vehicle system for controlled release of ciprofloxaxin drug: an ophthalmic application. OpenNano.

[30] Nikolić GS, Cakić MD, Glišić S, Cvetković DJ, Mitić ŽJ, Marković DZ. Study of green nanoparticles and biocomplexes based on exopolysaccharide by modern Fourier transform spectroscopy. Fourier Transforms-High-tech Application and Current Trends: InTech; 2017.

[31] Gorban IE, Soldatov MA, Butova VV, Medvedev PV, Burachevskaya OA, Belanova A (2020). l-leucine loading and release in MIL-100 nanoparticles. Int J Mol Sci.

[32] Berghian-Grosan C, Olenic L, Katona G, Perde-Schrepler M, Vulcu A (2014). L-Leucine for gold nanoparticles synthesis and their cytotoxic effects evaluation. Amino Acids.

[33] Gawali SL, Barick B, Barick K, Hassan P (2017). Effect of sugar alcohol on colloidal stabilization of magnetic nanoparticles for hyperthermia and drug delivery applications. J Alloys Compd.

[34] López-León T, Carvalho E, Seijo B, Ortega-Vinuesa J, Bastos-González D (2005). Physicochemical characterization of chitosan nanoparticles: electrokinetic and stability behavior. J Colloid Interface Sci.

[35] Mussi SV, Parekh G, Pattekari P, Levchenko T, Lvov Y, Ferreira LA (2015). Improved pharmacokinetics and enhanced tumor growth inhibition using a nanostructured lipid carrier loaded with doxorubicin and modified with a layer-by-layer polyelectrolyte coating. Int J Pharm.

[36] Gaspar DP, Serra C, Lino PR, Gonçalves L, Taboada P, Remuñán-López C (2017). Microencapsulated SLN: an innovative strategy for pulmonary protein delivery. Int J Pharm.

[37] Kho K, Hadinoto K (2010). Effects of excipient formulation on the morphology and aqueous re-dispersibility of dry-powder silica nano-aggregates. Colloids Surf A.

[38] Yu H, Teo J, Chew JW, Hadinoto K (2016). Dry powder inhaler formulation of high-payload antibiotic nanoparticle complex intended for bronchiectasis therapy: spray drying versus spray freeze drying preparation. Int J Pharm.

[39] Muhsin MD, George G, Beagley K, Ferro V, Wang H, Islam N (2016). Effects of chemical conjugation of L-leucine to chitosan on dispersibility and controlled release of drug from a nanoparticulate dry powder inhaler formulation. Mol Pharm.

[40] Lebhardt T, Roesler S, Uusitalo HP, Kissel T (2011). Surfactant-free redispersible nanoparticles in fast-dissolving composite microcarriers for dry-powder inhalation. Eur J Pharm Biopharm.

[41] Demoly P, Hagedoorn P, de Boer AH, Frijlink HW (2014). The clinical relevance of dry powder inhaler performance for drug delivery. Respir Med.

[42] Usmani OS, Biddiscombe MF, Barnes PJ (2005). Regional lung deposition and bronchodilator response as a function of β2-agonist particle size. Am J Respir Crit Care Med.

[43] Finlay WH. The mechanics of inhaled pharmaceutical aerosols: an introduction: Academic Press; 2001.

[44] Soong T, Nicolaides P, Yu C, Soong S (1979). A statistical description of the human tracheobronchial tree geometry. Respir Physiol.

[45] Lavorini F, Pistolesi M, Usmani OS (2017). Recent advances in capsule-based dry powder inhaler technology. Multidiscip Respir Med.

[46] Xu Y, Turan ET, Shi Z, Franzyk H, Thakur A, Foged C (2022). Inhalable composite microparticles containing siRNA-loaded lipid-polymer hybrid nanoparticles: Saccharides and leucine preserve aerosol performance and long-term physical stability. Front Drug Deliv.

[47] Kunda NK, Alfagih IM, Dennison SR, Somavarapu S, Merchant Z, Hutcheon GA (2015). Dry powder pulmonary delivery of cationic PGA-co-PDL nanoparticles with surface adsorbed model protein. Int J Pharm.

[48] Chani MTS (2022). Fabrication and characterization of chitosan-CeO2-CdO nanocomposite based impedimetric humidity sensors. Int J Biol Macromol.

[49] Haddrell AE, Lewis D, Church T, Vehring R, Murnane D, Reid JP (2017). Pulmonary aerosol delivery and the importance of growth dynamics. Ther Deliv.

[50] Motiei M, Aboutalebi F, Forouzanfar M, Dormiani K, Nasr-Esfahani MH, Mirahmadi-Zare SZ (2021). Smart co-delivery of miR-34a and cytotoxic peptides (LTX-315 and melittin) by chitosan based polyelectrolyte nanocarriers for specific cancer cell death induction. Mater Sci Eng C.

[51] Perry JL, Tian S, Sengottuvel N, Harrison EB, Gorentla BK, Kapadia CH (2020). Pulmonary delivery of nanoparticle-bound toll-like receptor 9 agonist for the treatment of metastatic lung cancer. ACS Nano.

[52] Wei L, Surma M, Gough G, Shi S, Lambert-Cheatham N, Chang J (2015). Dissecting the mechanisms of doxorubicin and oxidative stress-induced cytotoxicity: the involvement of actin cytoskeleton and ROCK1. PLoS ONE.

[53] Motiei M, Kashanian S, Lucia LA, Khazaei M (2017). Intrinsic parameters for the synthesis and tuned properties of amphiphilic chitosan drug delivery nanocarriers. J Control Release.

[54] Li J, Li B, Liu M (2019). One-step synthesis of mannose-modified polyethyleneimine copolymer particles as fluorescent probes for the detection of Escherichia coli. Sens Actuators B Chem.

[55] Mitchell JP, Nagel MW, Wiersema KJ, Doyle CC (2003). Aerodynamic particle size analysis of aerosols from pressurized metered-dose inhalers: comparison of Andersen 8-stage cascade impactor, next generation pharmaceutical impactor, and model 3321 Aerodynamic Particle Sizer aerosol spectrometer. AAPS PharmSciTech.

[56] Celluzzi A, Paolini A, D’Oria V, Risoluti R, Materazzi S, Pezzullo M (2018). Biophysical and biological contributions of polyamine-coated carbon nanotubes and bidimensional buckypapers in the delivery of miRNAs to human cells. Int J Nanomed.

[57] Indorf C, Dyckmans J, Khan KS, Joergensen RG (2011). Optimisation of amino sugar quantification by HPLC in soil and plant hydrolysates. Biol Fertil Soils.

